# Early impressions count: Category ratings established from small samples

**DOI:** 10.3758/s13414-026-03364-y

**Published:** 2026-10-28

**Authors:** Chuchen Zhang, Simon Kemp, Randolph C. Grace

**Affiliations:** 1 Department of Psychology, University of Canterbury, Christchurch, New Zealand; 2 School of Psychology, Speech, and Hearing, University of Canterbury, Private Bag 4800, Christchurch, 8140 New Zealand

**Keywords:** Category ratings, Categorization, Ensemble perception, Range-frequency theory

## Abstract

We examined the establishment of category ratings from small samples in three studies. A Monte Carlo experiment showed that basing categories on the first five or ten numbers encountered could provide a usable way to categorize numbers drawn from flat, normal and skewed distributions. Experiment 2 in which people categorized random integers presented one by one into five bins showed that after five or ten presentations the category structure was quite similar to that obtained after 30 presentations, and Experiment 3 found a similar result when people categorized arc segments. Overall, the results show that usable category ratings can be established from relatively small samples of observations.

**Supplementary Information:**

The online version contains supplementary material available at https://doi.org/10.3758/s13414-026-03364-y.

## Introduction

In this paper, we explore, via a Monte Carlo study and two experiments with participants, whether a useful categorization of stimuli ordered on a single dimension can be done with relatively few observations.

Categorizing is a basic human activity. Much theorizing in psychology has considered how people categorize stimuli that differ on a variety of dimensions (e.g., Anderson, 1991; Goodman et al., 2008; Roads & Love, 2024; Wang, 2024). How, for example, do people integrate multiple visual and auditory dimensions – such as body size, ear shape, facial contour, fur texture, gait, and even sound – to infer that a particular creature is a “cat”? Researchers have also studied the simpler case in which categories are formed on a single dimension (e.g., Seitz et al., 2025; Tsai et al., 2025). The single dimension might itself be made up from other components, for example, the excellence of wine might be determined by its smell, acidity, tannin content, etc., or it might be strictly unidimensional, for example, the magnitude of a number. In this paper we focus on strictly unidimensional categorization using numerical values or arc segments as stimuli.

The categories people employ are often made explicit, and visible to others, through category ratings. Although in psychology we often ask participants to categorize using numbered category ratings, category ratings do not have to be numerical, and many languages encode, for example, the information that someone is very tall, tall, average, short, or very short. Category ratings are thus extremely widespread, both in the everyday world and in psychological measurement. Parducci (1982, p. 89) considered them “direct pipelines to the psyche”. Their ubiquity signals that people find them useful.

Parducci’s (1965) range-frequency theory suggested that categories created on a single dimension are formed from a compromise of the range of stimuli to be categorized and their frequency distribution on the dimension. Subsequent experimentation has verified that categorization is indeed influenced by both these variables (e.g., Kemp et al., 2021; Parducci & Perrett, 1971; Parducci & Wedell, 1986). If we were to consider Parducci’s range-frequency theory, or something similar, to be an account of how people establish categories (rather than just the result), then categories would be formed after people have perceived and remembered many stimuli that are ordered on the dimension. When enough exemplars have been accumulated, reasonable estimates of the range and distribution of the stimuli could then be used to form either prototypes for each category class or boundaries between the classes.

However, a little consideration indicates that category ratings are probably not formed after painstaking accumulation of exemplars. On the theoretical side, accumulation implies a need to remember many exemplars. Moreover, until the accumulation is complete, category rating would not be possible. Empirical findings also lead to doubt. When children recall event or object classes, they appear to generalize quickly rather than remember specific details (Bloom, 2000; Nelson, 1974). There is also evidence that reasonably accurate prediction and concept formation do not always need many exemplars and that people may be ‘intuitive Bayesians’ in performing these tasks (Goodman et al., 2008; Griffiths & Tenenbaum, 2006; Vul et al., 2014).

Research into ensembles indicates that, given a class of stimuli, people tend to recall only summary statistics of the class (such as its mean and range) rather than individual stimuli (Ariely, 2001; Chong & Treisman, 2003; Cui & Liu, 2021; Frost et al., 2019; Whitney & Yamanashi Leib, 2018), and in Experiments 2 and 3 below, the results of ensemble research were used to decide the form of feedback to our participants. On the larger issue, these findings suggest that people may rely on summary statistics obtained from small samples to form category structures, although to our knowledge there has been little research into how small the samples might be.

At this point a question arises: What should the category structure be? Although this is clearly an important question, there does not seem to be a clear answer. In part this is because different categorization schemes might be useful in different circumstances. Probably for unidimensional stimuli the categories should be ordered: Ordering people’s heights as very tall, a little short, very short, average, a little tall is not useful. An initial categorization scheme that placed almost all later stimuli in a single category would also not be useful. Nor is a scheme in which some categories are never used at all.

However, the usefulness of a categorization structure depends on the stimuli, particularly their distribution. If, for example, the stimuli are uniformly distributed over some interval (e.g., a flat distribution on [0,1]) then, other things being equal, categories are probably more useful if they are equally spaced and filled. However, a bimodal distribution might result in placing most stimuli in categories two and four of five categories. If the distribution is normal, it might be useful to place more stimuli in the middle category than the extremes, and, if it is positively skewed, perhaps more stimuli should be assigned to the left-most category. Many different models of optimization could be suggested, but here we simply note that the categorization strategies may depend, as Parducci (1965) suggested, on the range and distribution of the stimuli.

We report three experiments which used different approaches to study the effectiveness of small-sample categorization. In Experiments 1 and 2, randomly generated numerical stimuli presented one at a time from flat, normal, or positively skewed distributions were assigned to five categories. Experiment 1 used a Monte Carlo method to compare the results obtained from three simple (perhaps simplistic) categorization algorithms with those obtained from more extensive sampling. We consider this experiment to provide a limited theoretical answer to the issue of how well small-sample categorization might perform. In Experiment 2, we asked participants to categorize the magnitude of integers as they perceived them. The distributions had different ranges as well as different distributions, so initially the participants could have little idea of what they would encounter. Experiment 3 was similar to Experiment 2 but presented arc segments, which necessarily have an upper bound (unlike integers). These two experiments were designed to address the issue of how the categorizing done over the first five or ten trials corresponds to categories obtained after 30 trials. For clarity hereafter, presented stimuli are always referred to as stimuli, integers, or arc segments, and the word number is reserved solely for the number of stimuli allocated to each category.

## Experiment 1

This study investigated the assignment of stimuli to five categories under different conditions via Monte Carlo simulation. There are many possible algorithms for how small-sample categorization could be carried out (e.g., Nosofsky & Stanton, 2005). We chose three simple algorithms that represented broader prototype-, boundary-, and range-based methods.

1. The *first five* stimuli encountered are taken as prototypes for five categories (Minda & Smith, 2011). Subsequent stimuli are then assigned to the closest prototype using simple linear distance.
2. The first four stimuli form *four* category *boundaries* (Parducci, 1965). Subsequent stimuli are then placed according to which category they fall into. Unlike for algorithms 1 and 3, there is no distance measure and simple ordering suffices.
3. The first *n* (5, 10 or 20) stimuli are sampled. The *highest and lowest* values are taken as the highest and lowest prototypes, and intermediate prototypes are evenly placed on the interval between them.

### Method

All stimuli – both during the formation stage and subsequently – were randomly generated from the underlying distributions. Because the effectiveness of categorization might depend on the type of the underlying distribution, each method – *first five*, *four boundaries* and the three *highest and lowest* methods – was investigated using flat, normal, and positively skewed distributions. The flat distribution was sampled from [0,1]. The normal distribution had mean 0, standard deviation 1. The skewed distribution was a truncated negative exponential, generated from a negative exponential distribution, *f(x) = λe*^*-λx*^, with rate parameter *λ* = 1/5 and retaining only values in the interval [0,1]. Note that statistics regarding the prototypes or boundaries are not directly comparable across distributions, although the number of stimuli placed in the categories are.

All simulations were performed in Python. The coding used is available on the Open Science Framework (OSF; https://osf.io/gphkw/) as are the data from Experiments 2 and 3. Each simulation examined how the method performed in sorting 30 stimuli into five categories (called bins below and throughout), repeated 5,000 times for each simulation. The key dependent variables we examined are the numbers of stimuli placed in each of the 5,000 × 5 bins.

`The small-sample categorization conditions were compared with control conditions for each method and distribution. The control conditions were obtained from averaging over 5,000 simulations for each small-sample categorization method and distribution using long-term means that differed with the small-sample categorization method as outlined below. The rationale for using a statistical procedure for the controls is that, even if the prototype or boundary means are known exactly, the number of stimuli placed in each bin for any sample of 30 stimuli varies. Given these means, 5,000 simulations of 30-sample bins were performed to obtain the control statistics.

*First five*. The first five stimuli generated were ordered to form the prototypes. Then a further 25 stimuli were also placed into the five bins (according to the linearly closest prototype) for a total of 30. This procedure was repeated 5,000 times. The prototype means for the control condition for the flat distribution were set as 0.1, 0.3, 0.5, 0.7, 0.9. However, for the normal and skewed distributions they were obtained by averaging the 5,000 means for each of the five bins. (The smallest control prototype was the mean of the 5,000 Bin 1 prototypes and so on.)

*Four boundaries*. The first four random stimuli were chosen and sorted as the inter-category boundaries (thus forming five categories). Thirty new random stimuli were obtained for each of 5,000 simulations, and these were slotted into the five categories using simple greater than and less than comparisons. For example, values less than the first boundary were assigned to Category 1, values between the first and second boundaries to Category 2, and so on. The control boundary means for the flat distribution were simply 0.2, 0.4, 0.6, and 0.8. For the normal and skewed distributions they were taken as the means of the 5,000 smallest boundaries and so on (a similar procedure to that used for the *first five* normal and skewed distributions).

*Highest and lowest*. For each simulation, a set of stimuli (*n* = 5, 10, or 20) was generated and the highest and lowest values selected as the prototypes for Bins 1 and 5. They also provided anchor values for the other three prototypes, which were calculated as the Lowest +.25,.50, or.75 × (Highest – Lowest). Thus, the central prototype was defined as the midpoint between the minimum and maximum values. Two intermediate prototypes were then defined as midpoints between the minimum and central prototype, and between the central and maximum prototype, respectively. Thirty new random stimuli were then assigned to bins according to the linearly closest prototype. Separate simulations were performed for each distribution and each initial sampling number. The controls differed across distribution types, and the three sets of means were obtained by averaging (over 5,000 simulations) the five prototypes obtained for the initial sampling of ten stimuli only.

The critical results refer to how the different bins are used for the small-sample categorization methods and how these compare to the control conditions. We chose the average absolute deviation, that is, the difference between the actual (small-sample and control) and the expected bin sizes to compare the performances of the different categorization methods across distributions. This measure took the number assigned to each frequency distribution, small-sample categorization method, and bin, and each of the 5,000 trials, and subtracted the expected average bin count for the appropriate control mean. For example, for the very first simulation run for the *first five* flat distribution condition, 9 (of the 30) numbers were placed in Bin 1 (that with the lowest numbers). The average for the control mean is 6, so the absolute error was 3. These values were then averaged to obtain what we call the average absolute error for each combination of conditions. Control condition errors were similarly calculated, that is, for each of the control simulation iterations the absolute difference between the long-term expected mean and that obtained from the 30 control samples was calculated. These control condition errors indicate how variable the categorization methods are compared to what would happen if the computer ‘knew’ the distributions obtained from very-long term sampling.

### Results

**Fig. 1:**
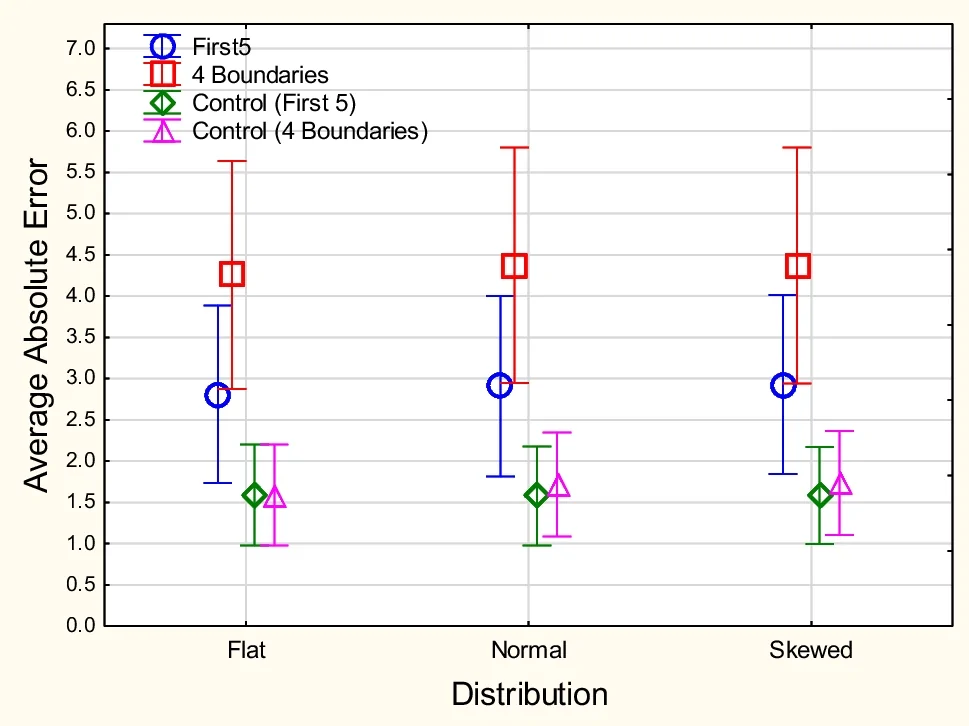
Absolute bin-size error averaged over 5,000 simulations and five bins for the three distributions for the first five, four boundaries, and their Control conditions in Experiment 1. Standard deviations of the absolute errors are also shown

**Fig. 2:**
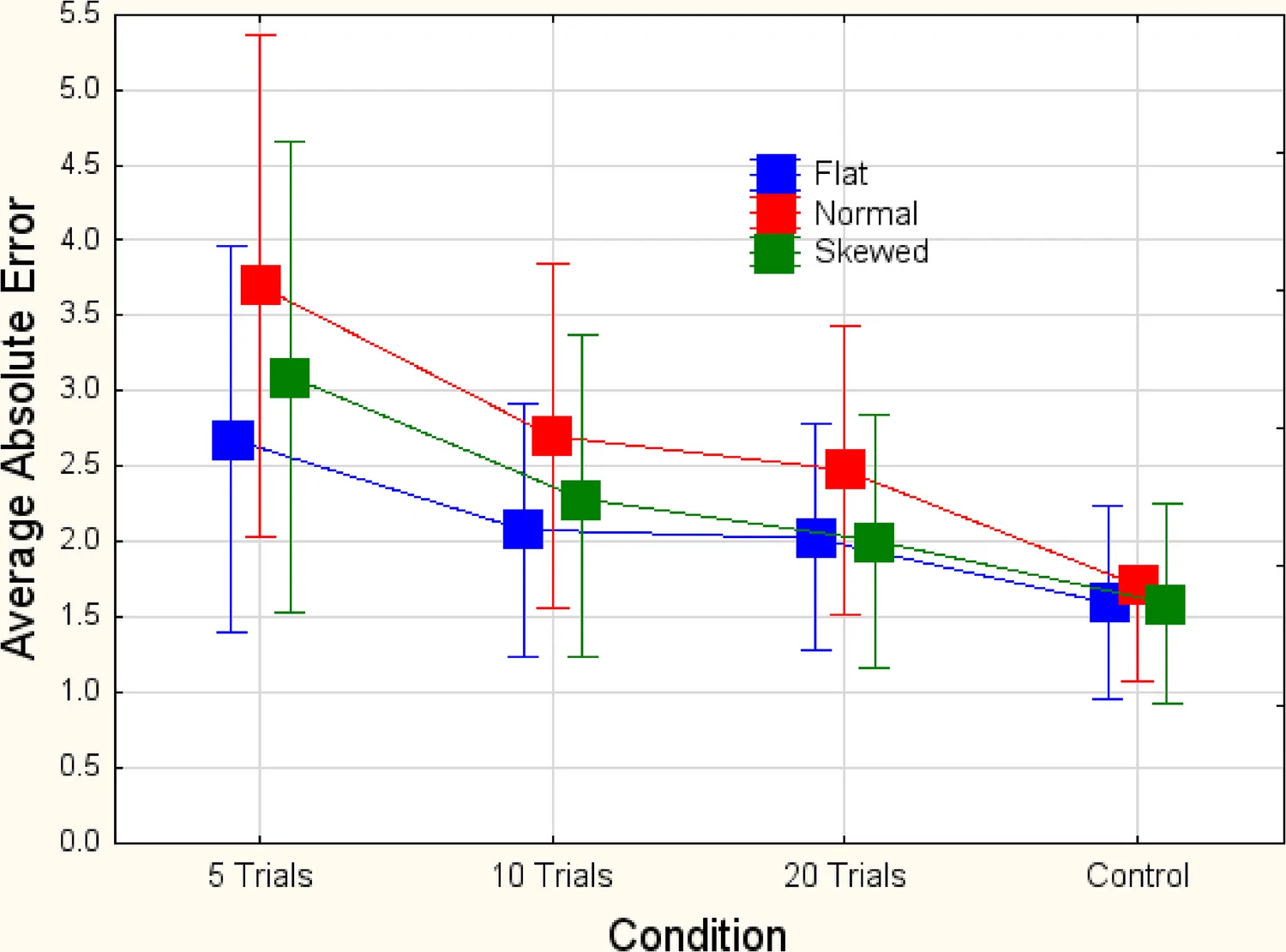
Average absolute bin-size error over 5,000 simulations and five bins for three conditions in which the highest and lowest prototypes are selected from the 5, 10, or 20 trials sampled in Experiment 1. The control condition is based on the average prototypes for the “10” condition. Standard deviations of the absolute errors are also shown

The results of the average absolute error comparisons for the *first five* and *four boundaries* categorization methods are shown in Fig. 1, and those for the *highest and lowest* categorization method in Fig. 2.

Figures 1 and 2 show that the *first five* method produces smaller absolute errors in bin size than the *four boundaries* method. (One way to understand this apparently odd result is that the cut points resulting from the *first five* method arise from averaging two prototypes rather than being completely unconstrained as in the *four boundaries* method.) Secondly, the *highest and lowest* method (for initial samples of 10 or more) produces less error than the *first five* or *four boundaries* methods. Thirdly, extending the initial sample from 10 to 20 for the *highest and lowest* method has little effect. Fourthly, the distribution does not matter much.

Finally, small-sample categorization produces larger errors than the control conditions for any method chosen. The *first five* method produces an average error that is not quite double the error produced from the control condition (depending slightly on the frequency distribution). The *highest and lowest* ten-trial method produces an average error about 50% more than the control (depending somewhat on the frequency distribution).

Table 1 shows the average bin sizes for each small-sample categorization method and distribution. The *four boundaries* method gives more even results across the five bins, while the *first five* method assigns relatively more stimuli to the end bins. Bin 1 generally contains more stimuli for the skewed distribution, but Bin 3 does not have more stimuli for the normal distribution. Some other results obtained from Experiment 1 are given in the Online Supplementary Material.

**Table 1:** Mean number (with standard deviations in parentheses) of placements in each bin in Experiment 1 for each distribution type and the first five, four boundaries, and highest and lowest categorization methods

Bin:	1	2	3	4	5
**Flat distribution [0,1]**					
*First five*	7.3 (4.2)	5.2 (2.8)	5.2 (2.9)	5.1 (2.8)	7.3 (4.2)
*Four boundaries*	6.0 (5.2)	5.9 (5.2)	6.1 (5.4)	6.0 (5.4)	6.0 (5.3)
*Highest/lowest* of 5	7.5 (4.5)	5.0 (2.4)	5.0 (2.4)	5.00 (2.5)	7.5 (4.4)
*Highest/lowest* of 10	5.7 (3.1)	6.2 (2.4)	6.1 (2.3)	6.2 (2.4)	5.8 (3.1)
*Highest/lowest* of 20	4.8 (2.4)	6.8 (2.4)	6.8 (2.3)	6.8 (2.4)	4.9 (2.4)
**Normal distribution (mean = 0; *SD* = 1)**					
*First five*	6.8 (4.3)	5.5 (3.3)	5.4 (3.1)	5.5 (3.3)	6.7 (4.2)
*Four boundaries*	6.0 (5.3)	6.1 (5.3)	5.9 (5.2)	6.0 (5.3)	6.0 (5.3)
*Highest/lowest* of 5	7.0 (5.2)	5.1 (2.9)	6.0 (3.3)	5.0 (2.9)	7.0 (5.2)
*Highest/lowest* of 10	4.6 (3.7)	6.3 (3.1)	8.2 (3.2)	6.3 (3.0)	4.6 (3.7)
*Highest/lowest* of 20	3.0 (2.7)	6.9 (3.2)	10.0 (3.2)	7.0 (3.2)	3.1 (2.7)
**Skewed (λ = 1/5)**					
*First five*	7.6 (4.3)	5.4 (3.1)	5.3 (3.1)	5.4 (3.4)	6.4 (4.2)
*Four boundaries*	6.2 (5.4)	5.9 (5.3)	6.0 (5.3)	6.1 (5.4)	5.7 (5.3)
*Highest/lowest* of 5	10.6 (5.0)	7.0 (3.3)	4.0 (2.1)	2.5 (1.7)	5.8 (5.0)
*Highest/lowest* of 10	10.7 (4.1)	9.1 (3.0)	4.5 (2.2)	2.4 (1.7)	3.3 (3.3)
*Highest/lowest* of 20	11.6 (3.9)	10.3 (2.7)	4.4 (2.1)	2.0 (1.6)	1.8 (2.1)

Apart from rounding error, all rows sum to 30

To summarize, the small-sample categorization methods, especially the *first five* and *highest and lowest* methods, did produce category rating structures, although with greater variability than very long-term sampling. These two methods also often produced uneven category use. The highest and lowest method suggests that a ceiling on the error was obtained after ten observations.

## Experiment 2

This study explored how people placed 30 integer stimuli presented one at a time into five categories or bins. As the experiment progressed, participants saw the updated average of the stimuli placed in each of the bins and the number of integers placed in each bin. The choice of feedback here followed, and thus used, the results from ensemble research discussed above. However, our provision of these data means that participants could access more precise results than they might have obtained on their own. Alternatively put, in this experiment the working out of the average and bin size results was controlled, while in most ensemble research it is not. The stimuli presented were drawn from flat, normal, and skewed distributions. Participants were instructed to try to order the averages from smallest (left bin) to largest (right bin). Otherwise, how the participants categorized – unlike in the Monte Carlo study – was left to them.

### Method

In a pilot study, six participants were given the option, after ten and 20 trials, to merge two bins and create a new one at the place of their choice, and to swap the positions of adjacent bins (e.g., they might make Bin 4 into Bin 5 and Bin 5 into Bin 4; Tsai et al., 2025). The pilot participants found merging and new bin creation difficult, so in Experiment 2 this option was excluded, but the possibility of bin swapping was retained. This possibility allowed participants to build up initial categories that might not be well ordered and then order them later.

#### Participants

Twenty-seven participants (nine male, 17 female, one non-binary; age range from 21 to 60 years, mean 30 years), were recruited. All participants reported normal or corrected-to-normal vision. Recruitment was conducted online using social media, and participants received a $10 voucher after completion of the experiment. The research was approved by our university’s Human Research Ethics Committee.

#### Procedure

Participants were tested individually. They sat at a desk using a C018EW HP Elite Mini 800 G9 Desktop computer with a standard LCD monitor with a resolution of 1,680 × 1,050 pixels and a maximum brightness of 300 cd/m^2^, and contrast ratio of 1000:1. The task was programmed using PsychoPy (PsychoPy Coder v2025.1.1, Python).

**Fig. 3:**
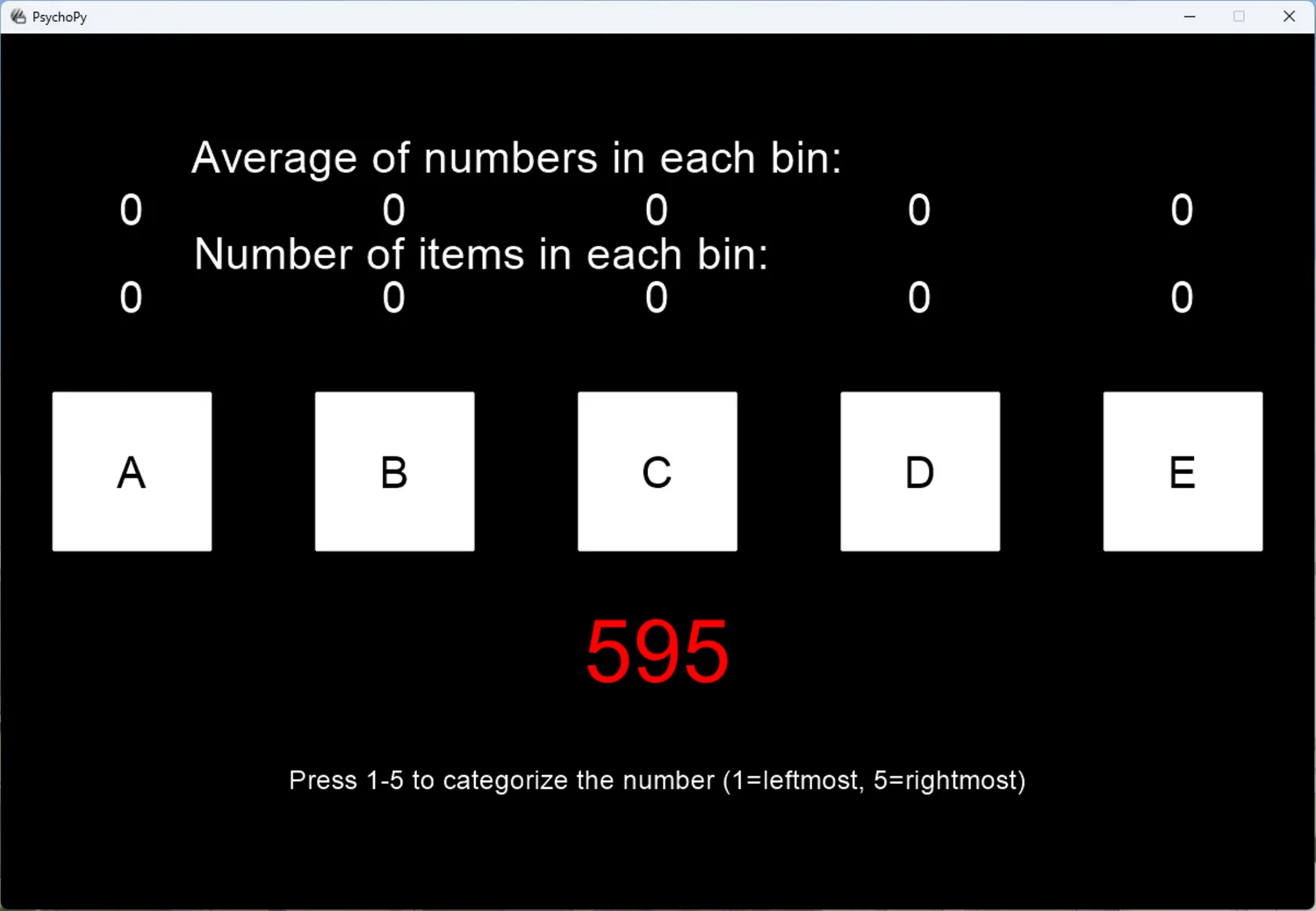
Illustration of categorization page of one trial in Experiment 2

Five white square bins (ABCDE) were displayed horizontally at the center of the screen (0.18 × 0.18 normalized units). Above each bin, the current mean and count of assigned stimuli were updated in real time after participant responding and reset every 30 trials. In each trial, a red integer stimulus was shown below the bins. Figure 3 illustrates the display. Participants were asked to try to ensure that the final categories were ordered from lowest integers on the left to highest on the right.

A total of 270 stimuli were randomly presented across nine distribution conditions (30 trials per condition), including three flat (uniform) distributions (U(0–40), U(0–200), U(0–1000)), three normal distributions (N(20, 5), N(100, 25), N(500, 100)), and three positively skewed negative exponential distributions (Exp(mean =20), Exp(mean =100), Exp(mean =500)) that used Python’s random.expovariate (1/mean) function, *f(x) = λe*^*-λx*^, which corresponds to rate parameters λ = 0.05, 0.01, and 0.002 respectively. Stimuli were randomly drawn without replacement within each condition.

In each trial, participants were required to categorize the presented integer into one of the five bins, using keys 1–5 corresponding to A–E for their responses. Before starting, participants read the task instructions and confirmed their understanding. There was no practice phase, and no reaction time limit. After a response was made, the current stimulus disappeared immediately, the appropriate mean and count updated, and the next stimulus presented until all 30 trials in the condition were completed. The average and number in the five bins were reset to zero at the start of each condition. Participants were informed before each new condition that the distribution would change. The order of the conditions was randomly determined and differed between participants. In each condition, at any time after ten trials had elapsed, participants could swap the positions of any two bins if they desired. The entire experiment took approximately 20 min to complete.

### Results

**Fig. 4:**
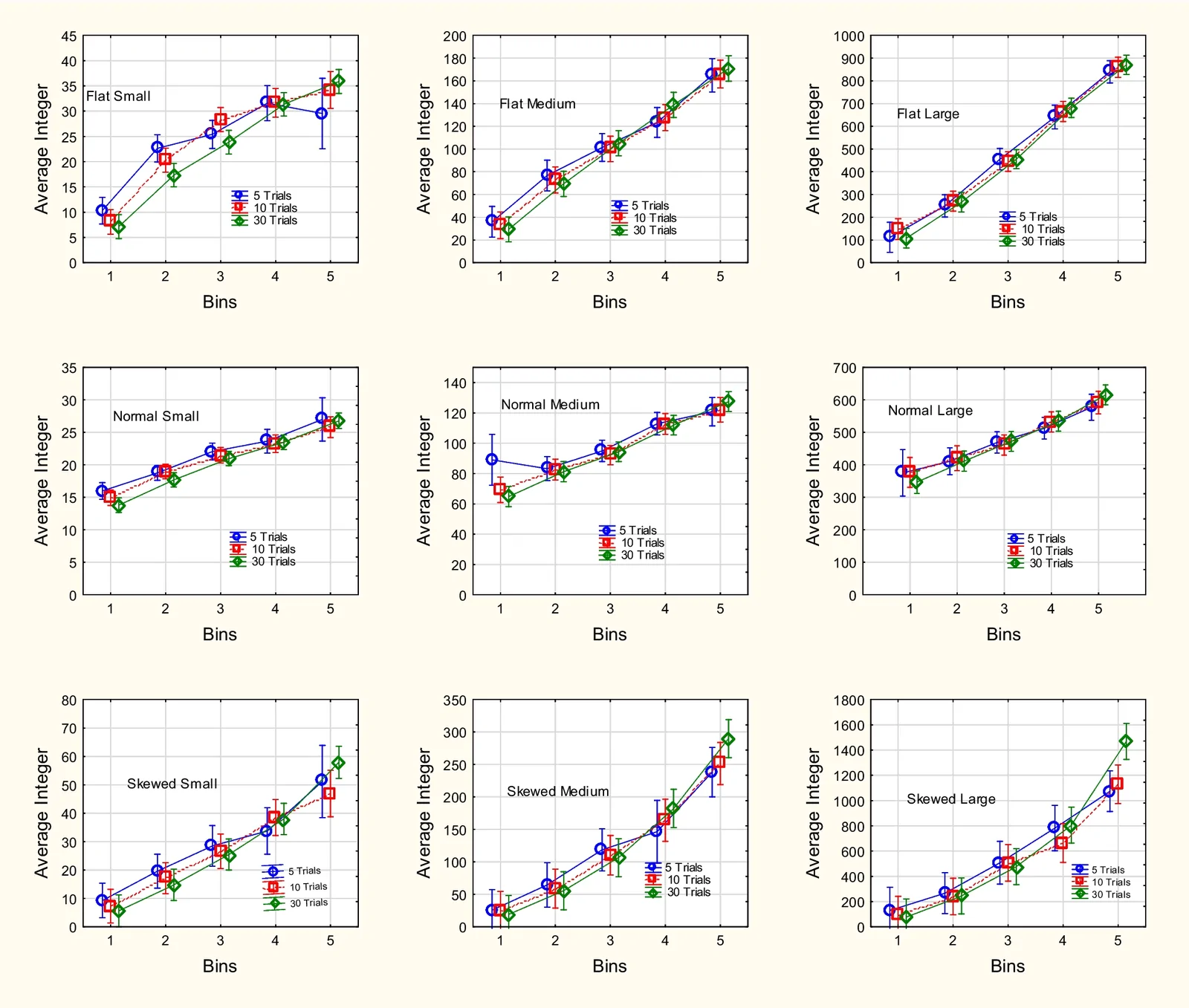
Integer stimuli averaged over 27 participants (with 95% confidence intervals) in each bin after 5, 10, and 30 trials for each of nine conditions, 3 (flat, normal and skewed distributions) × 3 (small, medium, and large integer stimuli presented) in Experiment 2. Where a participant placed no number in a bin after trial n, the data for that participant, trial and condition were missing (see text for more detail)

Figure 4 shows the average of the integer stimuli placed in each of the five bins for each distribution and the three stimulus ranges employed for each distribution. The averages after five, ten, and 30 trials were generally quite similar. Many participants did not use all the bins, and such results were missing data in Fig. 4.

**Fig. 5:**
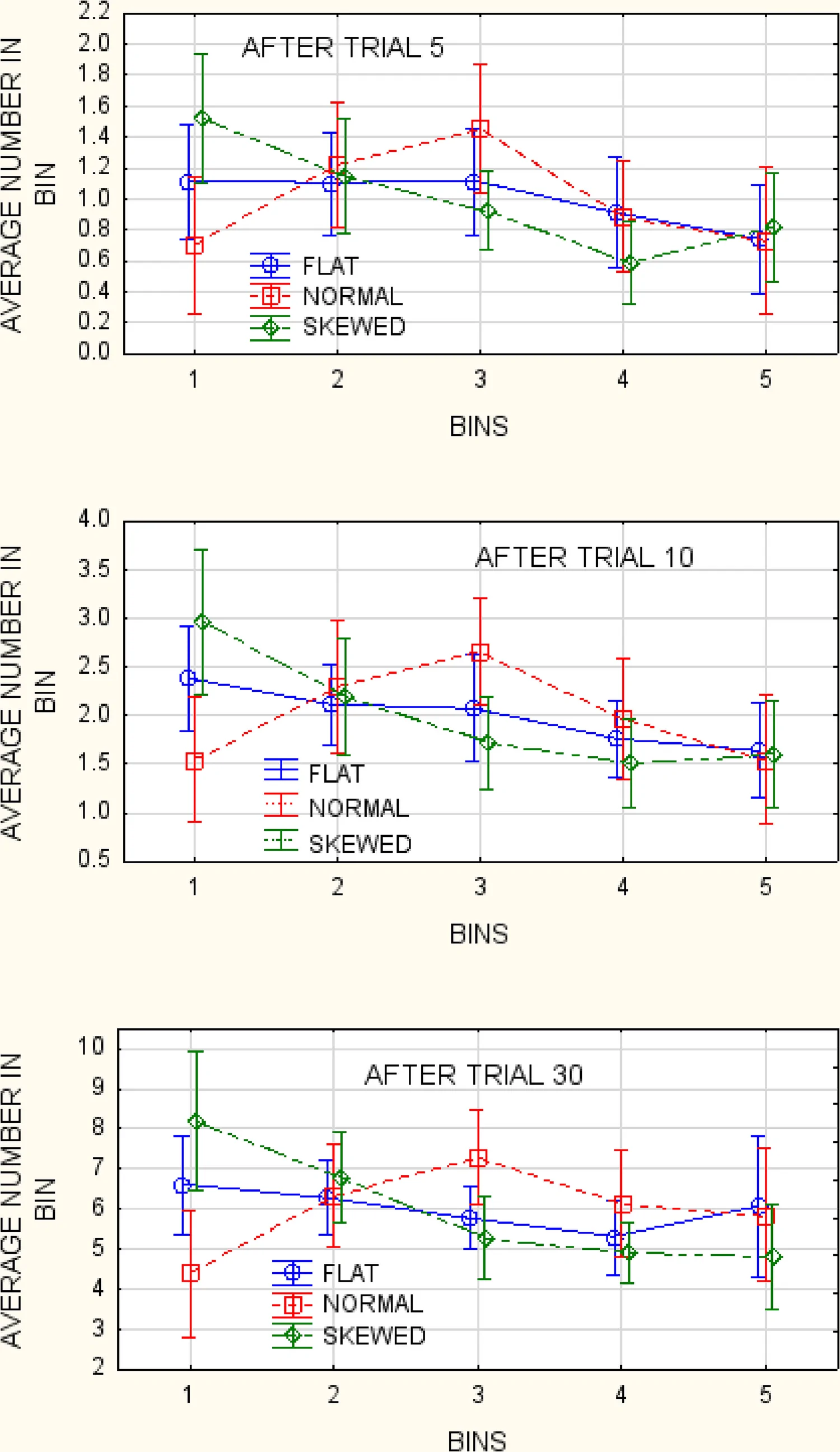
Numbers of integer stimuli placed in each bin after 5, 10, and 30 trials for each distribution averaged over the three integer sizes and 27 participants in Experiment 2 (95% confidence intervals are also shown)

Figure 5 shows the average number of integers placed in each bin after five, ten, and 30 trials. Participants tended to place more in Bin 3 for the normal distribution, and more in Bin 1 for the positively skewed distribution, even after five trials: Analysis of variance showed significant bin × distribution interaction effects after five (*F*(8, 208) = 5.51, *p* <.0001), ten (*F*(8, 208) = 7.01, *p* <.0001), and 30 trials (*F*(8, 208) = 10.91, *p* <.0001). Thus, even after only five integers had been observed, participants’ responses varied with the distribution.

Almost all the participants and conditions featured at least one empty bin after five trials (234/243 = 27 participants × 9 conditions), and only nine occasions saw the first five trials evenly distributed across the bins. After ten trials, 137(/243) still featured at least one empty bin, but after 30 trials, only 19 (/243) featured empty bins. The number of empty bins for individual participants (summed over the nine conditions, five bins, and three trial measures) ranged between 14 and 36 (mean = 24.6, *SD* = 6.1). To summarize, keeping bins unused for a time was a common strategy that did not vary much between participants.

Seventeen participants swapped bins at least once, with one participant producing five swaps. In total, there were 31 swaps, 22 occurring between trials ten and 19, and nine from trial 20 or later.

The experiment effectively prohibited participants from using the *four boundaries* method of Experiment 1, and made it difficult to use the *first five* method — recall that only 9/243 instances of the five-trial condition featured an even distribution — but using the *highest and lowest* method for the five- and particularly ten-trial conditions was quite possible. Moreover, Fig. 4 shows the relatively even spacing of integer averages predicted by this method for most conditions. However, the *highest and lowest* method required a participant to assign integers to Bins 1 and 5 quite early. Figure 5 does not suggest participants often did this. A more rigorous test is to consider when at least one integer was assigned to both Bin 1 and Bin 5. In fact, only 46/234 of the five trials met this provision, and still only 129/243 after ten trials. Moreover, on average more Bins 1 and 5 were empty (47%) after five trials than the middle bins (30%; two-tailed test of proportion, *p* <.0001). To summarize, the participants, even after five or ten trials, often seemed to be applying other methods of categorization than the simple algorithms examined in Experiment 1.

**Fig. 6:**
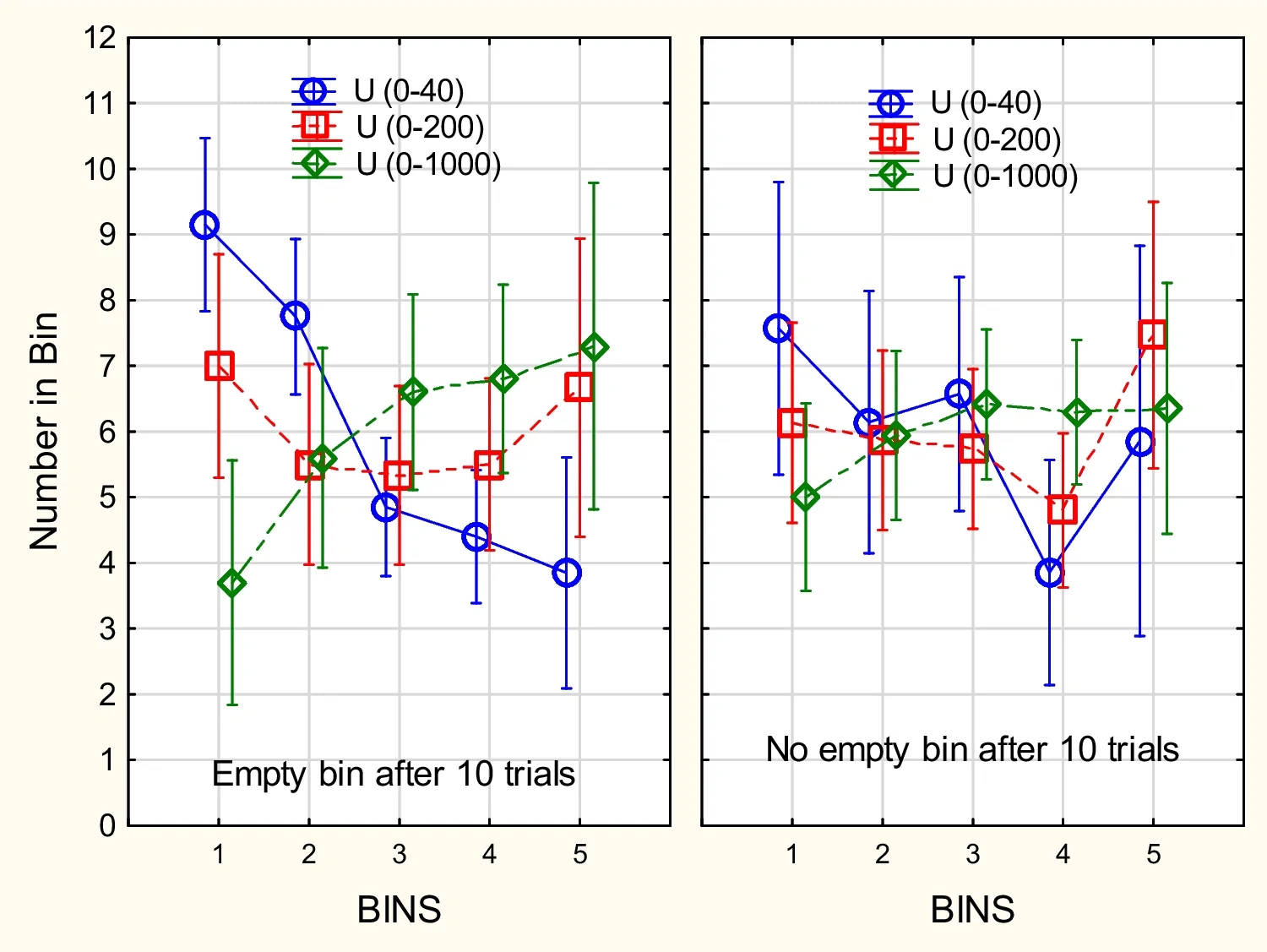
Average (with 95% confidence intervals) number of integers assigned to each bin after 30 trials for the three flat (uniform) distributions in Experiment 2. The left-hand graph shows results for when there was an empty bin after ten trials, and the right-hand graph shows results for when there was no empty bin. Results from 27 participants

As commented on in the *Introduction*, we might expect integers to distribute evenly across the bins for the flat distributions. Figure 5 shows that within the 95% confidence intervals there were even distributions for all bins, although the average number assigned was lower for Bins 4 and 5, and higher for Bins 1 and 2. The difference may reflect carry-over effects from previous conditions with high or low integers. For the flat distributions only, we also examined whether holding a bin empty by Trial 10 (42/81 instances) affected average bin placement after 30 trials, but we found no significant effects (Bins × Empty? interaction effect, *F*(4,300) =.59, *p* =.67; Bins × Empty? × Range interaction effect, *F*(8, 300) =.92, *p* =.50). Figure 6 illustrates the result, as well as carryover effects: Initially, participants place too few integers in Bins 1 and 2 and too many in Bins 4 and 5 for the 0–40 distribution; the opposite result holds for the 0–1,000 distribution.

The preceding analyses focus on data averaged over participants. Correlations of the numbers of integers placed in the bins obtained over the participants, distribution types, and stimulus sizes (see the upper panel of Table 2) show that the placement pattern from the first five trials tended to persist until the thirtieth trial.

**Table 2:** Pearson correlations between trials 5 and 10, 10 and 30, and 5 and 30 of numbers of stimuli assigned to each bin in Experiments 2 and 3

Trials:	5 and 10	10 and 30	5 and 30
**Experiment 2**			
Bins			
1	.83	.80	.62
2	.76	.67	.52
3	.74	.54	.40
4	.72	.60	.38
5	.81	.68	.51
**Experiment 3**			
Bins			
1	.83	.81	.65
2	.83	.81	.70
3	.82	.77	.62
4	.89	.85	.76
5	.89	.84	.74

All correlations differ from zero, *p* <.05

Bins with no integers assigned (number = 0) are included. Correlations for Experiment 2 are all based on 243 (3 × 3 × 27) observations, and those for Experiment 3 on 234 observations

## Experiment 3

Experiment 2 indicates that participants were able to establish categories from a limited number of stimuli (five or ten) and that the categorization scheme was often similar to that obtained after seeing a larger number of stimuli (30). One might ask whether these results would hold for other stimuli. Moreover, Experiment 2 provides few indications of how they did it. The very simple algorithms investigated in Experiment 1 were rarely used. Bin swapping was occasionally practiced by some participants but not others. It may be that a variety of different strategies can be used that vary partly between participants and partly with the experimental context. For example, both the conditions operating in the previous 30-trial block and the sheer chance of which integers within a condition the participant was first presented were probably important. It is also likely that in other circumstances a more systematic stimulus constraint is important. In Experiment 3 we investigated the effect of introducing such a stimulus constraint – an upper limit on the stimuli – by asking people to categorize segments of an arc, which are necessarily bounded below by 0 and above by 360 degrees.

### Method

#### Participants

Twenty-six participants (seven male, 19 females; age range from 19 to 61 years, mean 30.8 years), with normal or corrected-to-normal vision, were recruited online using social media. They each received a $10 voucher for their participation. The research was approved by our university’s Human Research Ethics Committee.

#### Procedure

Procedures, display, and testing were identical to Experiment 2, except that the participants viewed a red arc segment (varying from 0 to 360 degrees) that was always symmetrical around 12 o’clock. The averages were of the degrees covered by the arc segments.

The nine conditions (30 trials per condition) included three flat (uniform) distributions (U(mean = 90, range = 0–180), U(180, 0–360), U(300, 240–360)), three normal distributions (N(mean = 90, *SD* = 45), N(180, 45), N(300, 45)), with the sampled values truncated to ensure all generated stimuli remained within the range of a circle (0 to 360 degrees). Any sampled value less than 0‬ degrees‬ was set to 0‬, and any value greater than 360‬‬ degrees was set to 360. The three skewed negative exponential distributions (Exp(mean = 90, λ = 1/90), Exp(180, λ = 1/180), Exp(300, λ = 1/300)) shared an identical baseline (originating at 0‬) but had varied decay rates.‬ As for the normal distribution, the sampled values were bounded within [0, 360]. Note that for the mean = 300 condition, the distribution had a very flat decline, and a substantial proportion of the values mathematically exceeded 360‬ degrees, and because of truncation there was a spike of trials presenting an exact 360‬-degree circle.‬‬‬‬‬‬‬‬‬‬‬‬‬‬‬‬‬‬‬‬‬‬‬‬‬‬‬‬‬‬‬‬‬‬‬‬‬‬‬‬‬‬‬‬‬‬‬‬‬‬‬‬‬‬‬‬‬‬‬‬‬‬‬‬‬‬‬‬‬‬‬‬‬‬‬‬‬‬‬‬‬‬‬‬‬‬‬‬‬‬‬‬‬‬‬‬‬‬‬‬‬‬‬‬‬‬‬‬‬‬‬‬‬‬‬‬‬‬‬‬‬‬‬‬‬‬‬‬‬‬‬‬‬‬‬‬‬‬‬‬‬‬‬‬‬‬‬‬‬‬‬‬‬‬‬‬‬‬‬‬‬‬‬‬‬‬‬‬‬‬‬‬‬‬‬‬‬‬‬‬‬‬‬‬‬‬‬‬‬‬‬‬‬‬‬‬‬‬‬‬‬‬‬‬‬‬‬‬‬‬‬‬‬‬‬‬‬‬‬‬‬‬‬‬‬‬‬‬‬‬‬‬‬‬‬‬‬‬‬‬‬‬‬‬‬‬‬‬

### Results

**Fig. 7:**
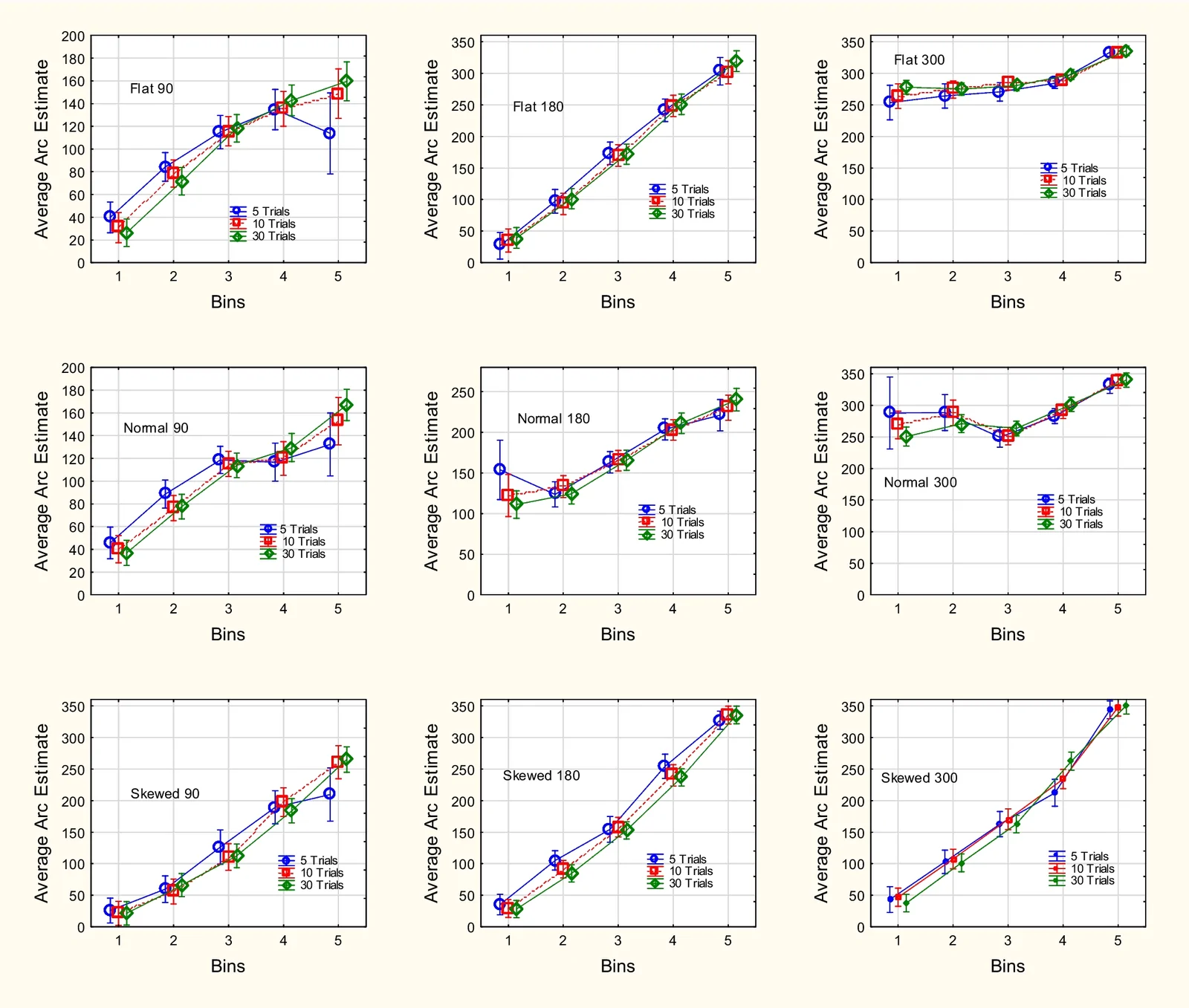
Arc segment estimates averaged over 26 participants (with 95% confidence intervals) in each bin after 5, 10, and 30 trials for each of nine conditions, 3 (flat, normal and skewed distributions) × 3 (small, medium, and large integer stimuli presented) in Experiment 3. Where a participant placed no number in a bin after trial n, the data for that participant, trial, and condition were missing (see text for more detail)

Figure 7 shows the average arc segment placed in each of the five bins for each distribution and the three stimulus ranges employed for each distribution. As in Experiment 2, the averages after five, ten, and 30 trials were generally similar. Many participants did not use all the bins, with such results again missing data.

**Fig. 8:**
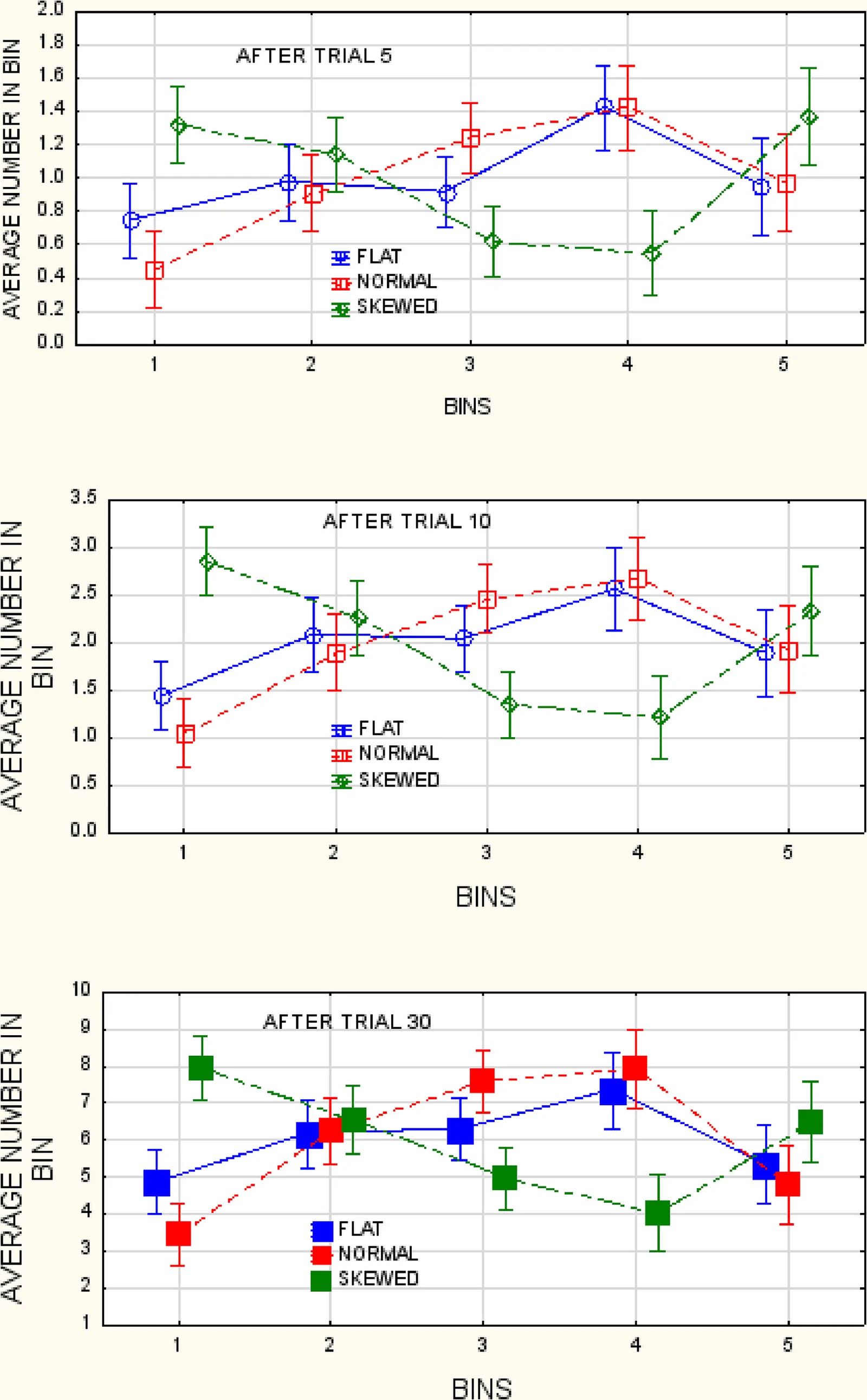
Numbers of arc segment stimuli placed in each bin after 5, 10, and 30 trials for each distribution averaged over the three integer sizes and 26 participants in Experiment 3 (95% confidence intervals are also shown)

Figure 8 shows the average numbers of stimuli placed in each bin after five, ten, and 30 trials. As in Experiment 2, participants placed more stimuli in Bin 3 for the normal and Bin 1 for the positively skewed distribution, and again analyses of variance found significant bin × distribution interactions after five (*F*(8, 200) = 8.04, *p* <.0001), ten (*F*(8, 200) = 9.51, *p* <.0001), and 30 trials (*F*(8, 200) = 10.49, *p* <.0001). Comparison of Fig. 8 with Fig. 5 indicates that in Experiment 3 relatively more stimuli were placed in Bins 4 and 5 than in the previous experiment, a result reflecting the asymmetric choice of arc segments presented in this experiment.

The remaining analyses focus on further differences from Experiment 2. The retention of empty bins persisted longer than in Experiment 2. There were 225 (/234) empty bins at the end of five trials, but 174 after ten trials and still 74 after 30 trials. The number of empty bins (summed over the nine conditions, three trial measures, and five bins) ranged between seven and 57 (average 35.0, *SD* = 12.4) for the 26 participants and was thus significantly (Mann-Whitney *U* = 157.5, *p* =.0006) greater than in Experiment 2. A similarly small proportion of stimuli (two-tailed test of proportion, *p* =.98), were placed in either Bin 1 or Bin 5 as in Experiment 2 after five trials (44/234), but after ten trials (103/234) the proportion was (just) significantly smaller (test of proportion, *p* =.049). In Experiment 3, only six participants performed swaps (with two participants doing it twice).

The lower panel of Table 2 shows the correlations of the numbers in each bin between trials five and ten, ten and 30, and five and 30 for Experiment 3. Note that for every cell but one, the correlations were higher (equal for Bin1, trails 5 and 10) than in the corresponding cell for Experiment 2.

## General discussion

In the simulation experiment (1), the *first five* and ten-trial *highest and lowest* algorithms produced absolute errors about double or 50% higher than when the parameters on which the algorithms were based were known exactly. Whether such increased errors are acceptable depends on the use and context to which the categorization is put, but in some contexts there might be a reasonable trade-off of this increased error for simplicity. The *four boundaries* algorithm produced a higher average absolute error but on average distributed the stimuli more evenly across the categories. On average, there was only a small reduction in absolute error for the *highest and lowest* method when the sample was increased from ten to 20 trials. In sum, Experiment 1 suggested that stable categorization might be possible from small samples of five to ten observations (e.g., Tenenbaum & Griffiths, 2001).

In Experiments 2 and 3 participants categorized stimuli that were either integers or arc segments from different distributions with different ranges. The data from both experiments – particularly the correlations of Table 2 and the generally similar average patterns of results for five, ten, and 30 trials shown in Figs. 4, 5, 7, and 8 – showed that categorization schemes adopted over the first five and ten trials often persisted to the 30th trial. Moreover, some features of normal and skewed distribution categorization were on average present in the first five trials in both experiments.

Overall, then, our results indicate that reasonable category ratings can be obtained from limited sampling when the feedback suggested from ensemble research (e.g., Whitney & Yamanashi Leib, 2018) is provided, and that about ten observations of random stimuli are needed to form five usable categories.

On the other hand, our research does not provide a clear indication of how the categories are obtained, and neither Experiment 2 nor Experiment 3 were designed to do this. None of the simple algorithms explored in Experiment 1 were widely used in Experiments 2 and 3. The *four boundaries* and *first five* methods were made very difficult by the experimental design, while the ten-trial *highest and lowest* method was excluded by the early categories produced by many participants. Indeed, the results suggest that on average early categorization focused more on filling inner categories than outer ones. However, one prediction of the *highest and lowest* method – that category means should be fairly evenly spaced – was approximately true for many of the conditions shown in Fig. 4 and some of those in Fig. 7.

It may be that the similarity of results for five, ten, and 30 sometimes reflected unwillingness to alter an early, unsatisfactory categorization scheme. However, participants were not forced to stay with their original schemes, and some participants, particularly in Experiment 2, swapped categories to obtain a later scheme that was better ordered. Moreover, unfilled categories in the first five trials were often filled subsequently, especially in Experiment 2.

Previous research (e.g., Goodman et al., 2008; Griffiths & Tenenbaum, 2006; Vul et al., 2014) indicates that people take prior expectations into account in small-sample categorization. Our research is in line with these findings, although our participants could also reset their expectations. The randomly ordered presentation of conditions and the sheer chance of which stimuli were presented early in Experiments 2 and 3, while in line with how participants might encounter stimuli outside laboratory settings, meant that such order effects were not readily analyzable here. Further experiments which systematically vary stimulus presentation orders would be useful in discovering how people build up categories from small samples. Such experiments might also test whether people make use of Bayesian probabilities in the task (e.g., Sanborn et al., 2010; Tenenbaum & Griffiths, 2001).

The difference in results between Experiment 2, where the stimuli had no upper bound, and Experiment 3, which had a clear upper bound, shows that our participants did make some use of prior knowledge in their categorization. The participants in these experiments also used different strategies, sometimes holding categories open for later use even after viewing ten stimuli. There was also variation in the use of swapping. Such results are in line with Seitz et al.’s (2025, p. 596) suggestion that different processes may be combined in a categorization strategy.

In the *Introduction* it was suggested that participants might choose to place more stimuli in inner categories for normally distributed stimuli and in categories with smaller means (Bins 1 and 2) for the positively skewed stimuli, and participants in both Experiment 2 and Experiment 3 tended to do this (see Figs. 5 and 8). Overall, the results suggest that categorization, even with small samples, reflects a compromise of range (where the categories are evenly spaced) and distribution (where the categories contain equal numbers), in line with Parducci’s (1965) theory.

The reader might notice that we have nowhere definitely answered the question of how good or useful a category structure based on small samples is. Experiment 1 does enable quantitative estimates of the error in small as compared to large samples. In Experiment 2 small sampling generally allocated stimuli evenly for a flat distribution, where this might be expected. However, this and the prediction that respondents might allocate more to the middle categories for a normal distribution were essentially weak predictions because there were no consequences for producing different categorizations. It seems likely that different categorization strategies and different sample sizes, with correspondingly different variabilities, might be employed in different real-world situations. Contrast, for example, someone casually rating the quality of goods and services, which is probably the most frequent present-day use of category rating, with the categorization scheme used by a medical practitioner attempting a diagnosis. Variability in the latter can have very real consequences, and we would not expect categories based on small samples to be adequate.

## Conclusion

To summarize, our results show that usable categories can often be formed from small samples, even when the range and distribution of the stimuli are initially unknown. Our research also suggests that different categorization strategies might be employed in different situations, partly in line with prior expectations.

## Supplementary Information

Below is the link to the electronic supplementary material.

Supplementary file1 (DOCX 41 KB)

## Data Availability

Experiments 2 and 3 via the OSF at: https://osf.io/gphkw/

## Code availability

Experiments 1, 2, and 3 via the OSF at: https://osf.io/gphkw/

## Declarations

### Open practices statement

The Python code for the simulation Experiment (1) as well as the complete data sets used in Experiments 2 and 3 are available via the Open Science Framework (OSF) at: https://osf.io/gphkw/
