# Supplementary material for: Early impressions count: Category ratings established from small samples
Source: Atten Percept Psychophys. 2026 Oct 28;88(8):211. doi: 10.3758/s13414-026-03364-y (PMC13719842; doi:10.3758/s13414-026-03364-y)
Supplement: Supplementary file 1 [file 13414_2026_3364_MOESM1_ESM.docx]

**Supplement. Some other simulation results from Experiment 1.**

Table A1 shows some statistics obtained for the normal distribution for the experimental and control conditions of the *first five* method. For this method and distribution, the number of stimuli assigned to the bin is somewhat higher for the extreme bins. The somewhat higher standard deviations and the lower (10 %) and upper (90 %) deciles of the bin distributions obtained indicate that fast categorisation is more likely to produce atypical categorisation.

In Tables A2 and A3, we present three statistics: the difference between the highest and lowest average bin sizes (1.3 for the fast and 0.1 for the control in Table A1), the median standard deviation (3.3 for the fast and 2.0 for the control in Table A1), and the median upper (90 %) decile (10 for the fast and 9 for the control in Table A1). Table A2 presents these summary statistics for the *first five* prototypes and the four boundary methods along with the control conditions. In summary, there is more variability (as measured by the median standard deviation and median upper decile) in both fast methods than the control condition. This variability is more pronounced for the four boundaries method than the first five method. The variability is little affected by the distribution type. However, when we look at a measure of bias (difference between the largest and smallest average bins), a different conclusion is suggested. Here the results with the *four boundary* fast method are comparable to the control condition and always less than the *first five* method, except for the skewed distribution where the *four boundaries* method has the smallest largest/smallest difference. (This is not surprising: The *four boundaries* method only uses ordinal statistics and is thus distribution free.)

Table 3A shows summary statistics for the *highest and lowest* methods for each initial sampling size. To summarise, even for the smallest initial sample size (5), the variability of the bin sizes, as measured by the median standard deviation and median 90 % is not much higher than for the control condition, although tending to decrease as the initial sample size increases. The results for the difference between the highest and lowest bin sizes are not here a good indication of bias. As the initial sample size increases the highest prototype becomes higher and the lowest prototype lower, so that there are correspondingly fewer stimuli placed in the outer bins. Furthermore, with the skewed distribution the bin with the highest prototype possesses few stimuli, especially if the initial sample size is large, and there are large differences between the number in this bin and that with the lowest prototype.

Finally, Table A4 shows the mean values (prototypes) obtained for the *first five* and three *highest and lowest* methods.

**Table A1**.

*Statistics obtained from 5000 simulations for the normal distribution and the first five method.*

___________________________________________________________________________

Prototypes

Fast Mean	-1.17	-.50	.00	.50	1.18

Fast SD	.68	.56	.54	.56	.66

Number (/30) placed in bins

Fast Mean	6.8	5.5	5.4	5.5	6.7

Control Mean	6.0	6.0	6.0	6.1	6.0

Fast SD	4.3	3.3	3.1	3.3	4.2

Control SD	2.0	2.0	2.0	2.0	2.0

Fast 10 %	2	2	2	2	2

Control 10 %	4	3	3	4	4

Fast 90%	13	10	10	10	13

Control 90%	9	9	9	9	9

___________________________________________________________________________

*Notes*

Prototype Control Means and SDs are not given because the means are constrained to equal the Fast ones and the SDs are 0.

10 %. On 10 % of the simulations the number assigned to this bin is less than or equal to the number shown.

90 %. On 90 % of the simulations the number assigned to this bin is greater than or equal to the number shown.

**Table A2**.

*Selected bin statistics from Experiment 1 for the first five and four boundaries methods along with the control (taken from the first five).*

___________________________________________________________________________

*First five*	*Four boundaries*	Control

Flat distribution

M_H_ – M_L_	2.1	.18	.06

Median SD	2.9	5.3	2.0

Median 90 %	9	14	9

Skewed distribution

M_H_ – M_L_	2.3	.50	2.4

Median SD	3.4	5.4	2.0

Median 90 %	10	14	8

Normal distribution

M_H_ – M_L_	1.3	.14	.15

Median SD	3.3	5.3	2.0

Median 90 %	10	14	9

___________________________________________________________________________

*Notes*

M_H_ – M_L_ The difference between the largest and smallest average bin size.

Median SD The median of the five standard deviations of the bin sizes

Median 90% Ninety percent of the bins are filled with a certain number of stimuli or fewer. This statistic is the median over the five bins.

**Table A3**.

*Selected bin statistics from Experiment 1 for the highest and lowest method for three initial sampling sizes (5, 10, 20) along with a control taken from averaging the prototypes taken from the initial sample of 10*.

___________________________________________________________________________

Initial 5	Initial 10	Initial 20	Control

Flat distribution

M_H_ – M_L_	2.5	.45	2.0	.06

Median SD	2.5	2.4	2.4	2.0

Median 90 %	8	9	10	9

Skewed distribution

M_H_ – M_L_	8.1	8.3	9.8	`	9.0

Median SD	3.3	3.0	2.1	2.0

Median 90 %	11	8	7	7

Normal distribution

M_H_ – M_L_	2.0	3.6	7.0	5.3

Median SD	3.3	3.2	3.2	2.3

Median 90 %	10	10	11	10

___________________________________________________________________________

*Notes*

M_H_ – M_L_ The difference between the largest and smallest average bin size.

Median SD The median of the five standard deviations of the bin sizes

Median 90% Ninety percent of the bins are filled with a certain number of stimuli or fewer. This statistic is the median over the five bins.

**Table A4**.

*Mean integer stimulus value placed in each bin in the simulation study for each distribution type and the first five and highest and lowest categorization methods*.

___________________________________________________________________________

Bin:	1	2	3	4	5

Flat distribution [0,1]

Control	.10	.30	.50	.70	.90

*First five*	.17	.33	.50	.66	.83

*Highest/lowest* of 5	.17	.33	.50	.67	.83

*Highest/lowest* of 10	.09	.30	.50	.70	.91

*Highest/lowest* of 20	.05	.27	.50	.73	.95

Normal distribution (Mean = 0; SD = 1)

Control	-1.17	-.50	0	.50	1.18

*First five*	-1.17	-.50	0	.50	1.18

*Highest/lowest* of 5	-1.15	-.57	0	.58	1.15

*Highest/lowest* of 10	-1.54	-.77	0	.76	1.53

*Highest/lowest* of 20	-1.87	-.94	-.01	.93	1.86

Skewed (λ = 1/5)

Control	.04	.09	.16	.26	.46

*First five*	.04	.09	.16	.26	.46

*Highest/lowest* of	5	.04	.14	.24	.35	.46

*Highest/lowest* of 10	.02	.16	.31	.45	.59

*Highest/lowest* of 20	.01	.19	.37	.54	.72

___________________________________________________________________________

*Note*. Controls all taken from the *first five* method.
